# Vitellogenesis in the Fruit Fly, *Drosophila melanogaster:* Antagonists Demonstrate that the PLC, IP_3_/DAG, PK-C Pathway is Triggered by Calmodulin

**DOI:** 10.1673/031.013.6801

**Published:** 2013-07-15

**Authors:** Bethany J. Brubaker-Purkey, Richard I. Woodruff

**Affiliations:** Department of Biology, West Chester University, West Chester, PA 19383, United States.

**Keywords:** gap junction, PLC/PK-C signaling, YP endocytosis

## Abstract

In *Drosophila melanogaster* M. (Diptera: Drosophilidae), a phospholipase-C to proteininase-C signal cascade leads to the endocytic uptake of yolk precursor molecules. The data suggest that *D. melanogaster* has a phospholipase-C/proteinkinase-C signaling pathway similar to that previously shown to be required for vitellogenesis in the milkweed bug, *Oncopeltus fasciatus* Dallas (Hemiptera: Lygaeidae). Calmodulin, derived from epithelial cells and transported to the oocytes via gap junctions, may trigger this pathway. To investigate this, a series of known antagonists to various elements of the pathway were used. W-7 (which prevents calmodulin binding to phospholipase-C), U-73122 (which prevents activation of phospholipase-C), verapamil (which blocks Ca^2+^ release by IP3), HAG (which blocks diacylglycerol), and staurosporine (which inactivates proteinkinase-C) were each shown to inhibit endocytosis, thereby blocking formation of nascent yolk spheres.

## Introduction

*Drosophila melanogaster* Meigen (Diptera: Drosophilidae) has become one of the most widely studied multi-cellular organisms on the planet. Unfortunately, the small size of adult *D. melanogaster* females precludes collection of adequate hemolymph for *in vitro* incubation of individual organs such as ovaries, or the ovarian follicles within them. This limitation can be overcome by using hemolymph from other unrelated insects, as shown in a recently published report (Brown et al. 2011).

The signature event of developing insect oocytes is the receptor mediated endocytic uptake by oocytes of hemolymph-born female specific proteins, which are the precursor molecules of yolk. This class of proteins has been generally referred to as vitellogenins (Vgs) (Pan et al. 1969). Because the yolk precursors of *D. melanogaster* are biochemically different from the yolk precursors of some other insects, the specific term used for them is “YP” (Minoo et al. 1985). Here, the designation of “Vg” is used in the original nonspecific manner where appropriate, and “YP” when referring specifically to these polypeptides from *D. melanogaster*. Within membrane bound yolk spheres, these precursors are converted into yolk protein (vitellin). This stage is thus referred to as “vitellogenesis,” and it is during this stage of follicular development that most of the cytoplasm and all of the yolk are deposited in the oocyte (Telfer 1975; King et al. 1985). While the synthesis, transport, and processing of Vgs/YPs have been extensively studied (c.f. reviews by Engelmann 1979; Hagedorn and Kunkel 1979; Raikhel et al. 1992; Giorgi et al. 1999), for several insects the mechanisms specifically regulating the onset and continuation of endocytic activity by the oocyte remain in question. In several species of insects, ingestion of food (often a blood meal) begins a cascade of largely hormonal events culminating in production of Vgs, their release into the hemolymph, and receptor mediated endocytosis into yolk spheres in the terminal oocyte of each ovariole, while younger oocytes are inhibited from acquiring Vgs.

However, in many species, both previtellogenic and vitellogenic follicles are simultaneously present and actively developing. The hormone-regulated system clearly could not function in the same manner in the multitude of insects in which ovarian follicles of several different stages develop simultaneously. Concerning the actual mechanisms regulating the endocytic process, juvenile hormone has been shown in Diptera to be the stimulus for assembling the endocytic complex (Tedesco et al. 1981; Raikhel et al. 1985). Diet, temperature, and other hormones also play a part (Richard et al. 1998; Soller et al. 1999; et al. 2001). Previtellogenic follicles of many species are exposed to these global signals of diet and hormone, yet are not stimulated to change their physiological state and begin uptake of Vgs. Thus, there remains an important question: what signals a previtellogenic oocyte to activate this complex, initiate endocytosis, and become a vitellogenic oocyte?

It has been demonstrated in the milkweed bug, *Oncopeltus fasciatus* Dallas (Hemiptera: Lygaeidae), that a diffusible signal (in the general sense of a substance that will allow an event or cascade of events to occur upon its arrival) passes through gap junctions from the epithelial cells to the oocyte, causing it to begin and maintain active endocytosis of Vgs (Anderson et al. 2001), and that similar systems exist for representatives of six different orders of insects (Waksmonskiet al. 2002). For *O. fasciatus* the signal molecule has been identified as calmodulin (CaM) (Brooks et al. 2004), the presence of which permits functioning of a signal cascade in which phospholipase-C (PLC) converts PIP_2_ into IP_3_ and diacylglycerol (DAG). IP_3_ causes the release of Ca^2+^, which, along with DAG, stimulates the proteinkinase-C (PK-C) required for endocytic uptake of Vgs (Brown et al. 2010). The present study has taken advantage of the discovery that *D. melanogaster* oocytes will endocytically incorporate Vgs from other species (Brown et al. 2011), and showed *in vitro* that epithelial cell-to-oocyte gap junctions are patent and can allow passage of CaM, and that this same signal cascade requiring the entry of CaM of follicle cell origin is utilized by *D. melanogaster* for endocytic uptake of YPs.

## Materials and Methods

### Animals

*Drosophila melanogaster* (Oregon Red) were raised at room temperature on standard *D. melanogaster* medium (Carolina Biological Supply Co., www.carolina.com). Ovaries with multiple stage-10 follicles were obtained in the following manner. Newly emerged flies were placed in fresh vials. On the third day, they were fed a dollop of yeast paste, and were dissected 24 hr later on day four (Tilney et al. 1996). Females were dissected in a physiological salt solution (PSS) consisting of 100 mM Na-glutamate, 25 mM KCl, 15 mM MgCl_2_, 5 mM CaSO_4_, and 2 mM sodium phosphate buffer (pH 6.9). This medium was developed to match the ionic strength (Van der Meer et al. 1983) and osmolarity of adult *D. melanogaster* female hemolymph (Singleton et al. 1994).

*Oncopeltus fasciatus* (Carolina Biological Company) were reared on cracked sunflower seeds in constant light at 30° C to stimulate egg production (Kelly et al. 1979). Mating females were selected, decapitated, and their legs were amputated. From the sites of leg wounds, 1 μL microcaps (Drummond Scientific Co., www.drummondsci.com) were used to collect Vg-rich hemolymph, which was immediately transferred to *D. melanogaster* PSS. This was done to provide a *D. melanogaster* PSS with hemolymph components that would support endocytic formation of *D. melanogaster* yolk spheres (Brown et al. 2011). Thus, endocytic uptake of proteins in the experiments reported here could involve both Vgs, and the YPs if their receptors are related as suggested by Hagedorn et al. (1998) and Bownes et al. (2002).

### Determination of oocyte endocytic activity

Newly dissected ovaries were placed in spot plate wells half-filled with hemolymph-free PSS for 30 minutes. During this time, all YPs present in the spaces between follicle cells or in the peri-oocytic space were either washed out or were fully incorporated into nascent yolk spheres (NYS). In the absence of YPs, endocytosis came to a stop. Follicles thus treated are henceforth termed “YPC” (YP-cleared). Experimental ovaries were then transferred into PSS, to which had been added one of the membrane permeant antagonists (Table 1), and incubated for 30 min to ensure that the antagonist had acted. Tissue was then transferred to PSS containing the antagonist, *O. fasciatus* hemolymph, and a small amount of dextran labelled with Texas-Red dye (Dexred, 10 kDa, Molecular Probes, Invitrogen, www.invitrogen.com). These ovaries were then incubated an additional hour, during which any NYS formed would fluoresce due to Dex-red taken up by non-specific fluid phase incorporation. Ovaries were washed in three changes of PSS, and ovarioles within each ovary were gently teased apart to allow viewing individual stage 10 follicles, placed in a glass bottomed 35 mm Petri dish, and examined with a fluorescence microscope to determine the presence or absence of NYS. Control follicles were treated in the same manner, but not exposed to any antagonist.

### Microscopy

Observations of fluorescent materials were made using an Olympus IX-71 inverted microscope (Olympus America Inc., www.olympusamerica.com) equipped with epi-illumination fluorescence optics with the proper dichromatic mirrors, excitation filters, and barrier filters for visualization of the fluorophores used. The objectives used for fluorescence were either a 40X A-Plan Apo, N.A.= 0.95, or a 20X LCPlanF1, N.A.=0.40 N.A. This microscope was also equipped with an Olympus 1X2-DSU spinning disk confocal unit. Images were captured with a Spot Xplorer cooled CCD camera (Diagnostic Instruments Inc., Sterling Heights, MI), with iVision software (BioVision Technologies, Inc., www.biovis.com) running on an Apple Macintosh G-5 computer (Apple Computer Inc., www.apple.com).

### Microinjections

Prior to impalement, follicles were treated for 1 to 2 min with 1 mg/mL collagenase (type 1A, Sigma-Aldrich, www.sigmaaldrich.com) to soften the basement membrane. Microinjections were made either iontophoretically for Lucifer Yellow-CH or by pulsed gas pressure using a Narishige FM-200 gas pressure system for AlexaFlour 488-labeled CaM. Pressure injections were adjusted so that the bolus was equal or less than ¼ of the volume of the oocyte being injected.

**Table 1.**
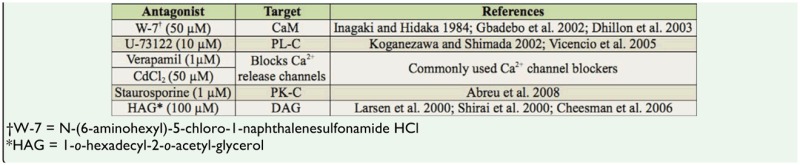
Antagonists, concentration used, target, references.

### Electrophysiology

Electrical measurements were made in a 0.45 mL, open-topped chamber grounded through an Ag/AgCl wire. Standard 3M KCl microelectrodes were attached to Electro 705 electrometers for membrane potential measurements, or to a Microiontophoresis Dual Current Generator 260 unit to provide current pulses (World Precision Instruments, www.wpiinc.com). Signals were stored on a Tektronix 5116 dual-beam storage oscilloscope with a 5D10 waveform digitizer (www.tek.com). Electrodes were carried by precision Chambers-type micropositioners (Line Tool Co., www.linetool.net), one of which was fitted with a Narishige MMO-203 hydraulic single axis micropositioner (Narishige, usa.narishige-group.com), for the final advance (Cieniewicz et al. 2008).

## Results

### Effect of antagonists upon the PLC-PK-C signal cascade:

As shown in earlier reports, by non-specific fluid phase uptake, the presence of Dex-red in the incubation media used in these experiments resulted in any NYS formed being fluorescent (Brown et al. 2010; Brown et al. 2011). The oblate spheroid shape of *D. melanogaster* follicles means that the surface of each follicle is curved. Thus, in optical sections such as those shown in this study, the center of any view revealed the deeper regions of the follicle, while more peripheral regions had in-focus structures progressively closer to the outer surface. In all of the micrographs presented here, the follicle epithelium cells at the periphery are at least partially in the plane of focus. Closer towards the center, the region of NYS formation can be seen. Focus at the center of the follicle is just beneath this level in the region where previously formed NYS clusters would eventually fuse into mature yolk spheres.

**Figure 1.**
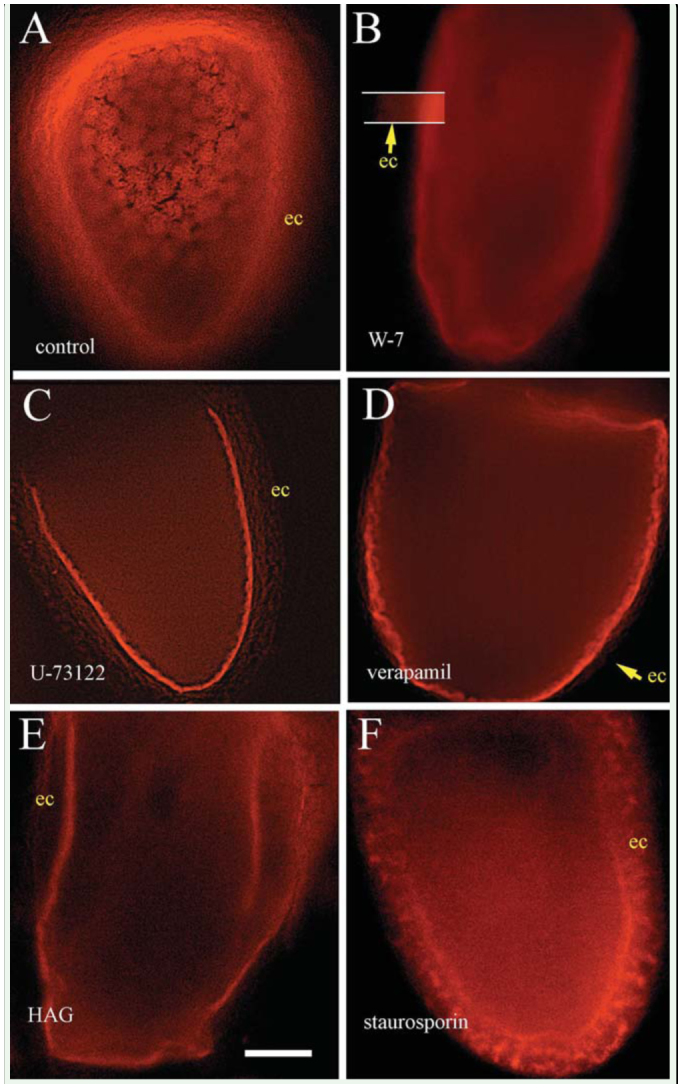
Spinning-disc confocal images of follicles of *Drosophila melanogaster* incubated in PSS-*Oncopeltus fasciatus* Vgs and Dexred. The Vgs stimulate endocytosis while the Dex-red, by nonspecific fluid phase uptake caused any nascent yolk spheres to fluoresce. Media around experimental follicles also contained antagonists of steps in the PLC/PK-C signal cascade leading to endocytic formation of nascent yolk spheres. Focus for each follicle was at the level within the ooplasm where clusters of nascent yolk spheres would form. 1A is a control follicle with clusters of fluorescent nascent yolk spheres. Follicles treated with antagonists of the PLC-PK-C signal cascade fail to complete endocytosis. Antagonists used were (1B) W-7, (1C) U-73122, (1D) verapamil, (1E) HAG, and (1F) staurosporine. Scale bar = 60 μm for all. ec = epithelial cells. High quality figures are available online.

Incubation of previously YPC *D. melanogaster* follicles in the presence of *D. melanogaster* PSS/Dex-red and Vg-containing *O. fasciatus* hemolymph resulted in the formation of NYS, clusters of which eventually fuse into mature yolk spheres. Figure 1A shows a confocal optical section of a control stage-10 follicle displaying multiple clusters of NYS. External to these, a narrow bright region marks the space between epithelial cells and oocyte surface. Most external are the epithelial cells. Video 1 focuses through the z-stack of this follicle.

When YPC follicles were incubated in this same manner, but with 50 μM W-7 added, endocytic formation of NYS was halted (Figure 1B). In Figure 1B, brightness and contrast have been increased in the small region between the bars to allow the very dim autofluorescence of the epithelial cells to be seen. Note that in the ooplasm there are neither individual nor clustered NYS. W-7 binds to CaM, keeping it from interacting with other molecules, in this case possibly with PLC (Inagaki et al. 1984; Gbadebo et al. 2002; Dhillon et al. 2003).

**Video 1.**
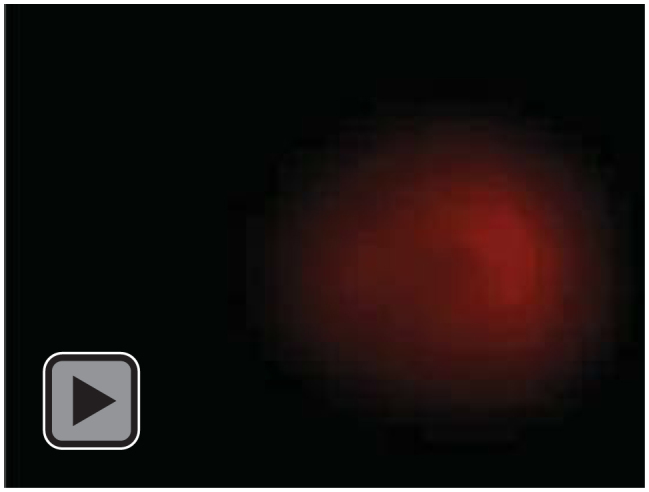
Spinning-disc confocal sequential Z-axis changes in focus through a control ovarian follicle from *Drosophila melanogaster*. As focus reaches deeper levels the apical end of epithelial cells are passed. Just inside the epithelial cells is a bright dyefilled space between epithelium and oocyte surface. At deeper focus, within the ooplasm, there are clusters of nascent yolk spheres. These will join together into mature yolk spheres. Mature yolk spheres at the center of the oocyte were formed before Dex-red was added and thus do not contain this dye. Click image to view video. Download video

The substrate of PLC is PIP_2_ (phosphatidylinositol 4,5-bisphosphate), and the products are IP_3_, which is known to stimulate release of Ca^2+^ from the endoplasmic reticulum, and DAG, which is known to directly stimulate the enzyme PK-C. U-73122, the antagonist of PLC (Koganezawa et al. 2002; Vicencio et al. 2005) employed in the present study, terminated formation of NYS (Figure 1C), linking PLC to the CaM-initiated signal cascade.

An increase in [Ca^2+^]_i_ is known to stimulate PK-C. IP_3_-timulated release of Ca^2+^ from internal sequestration can be inhibited by Ca^2+^ release channels blockers such as verapamil or CdCl_2_. Verapamil at 1 μM, or 50 μM CdCl_2_, caused termination of NYS formation (Figure 1D; CdCl_2_ data not shown). In Figure 1D, the epithelial cells are so dimly autofluorescent that they cannot be seen. The arrow marks their outer edge. HAG binds to DAG, disenabling it from binding to other molecules, including PK-C (Larsen et al. 2000; Shirai et al. 2000; Cheesman et al. 2006). HAG at 100 μM stopped formation of NYS (Figure 1E). Finally, staurosporine is a specific antagonist of the enzyme PK-C (Abreu et al. 2008). Inclusion in the medium of 1 μM staurosporine stopped all formation of NYS. This is shown in Figure 1F and Video 2, which focuses through the z-stack of this follicle. Results of each of the antagonists are summarized in Table 2.

**Video 2.**
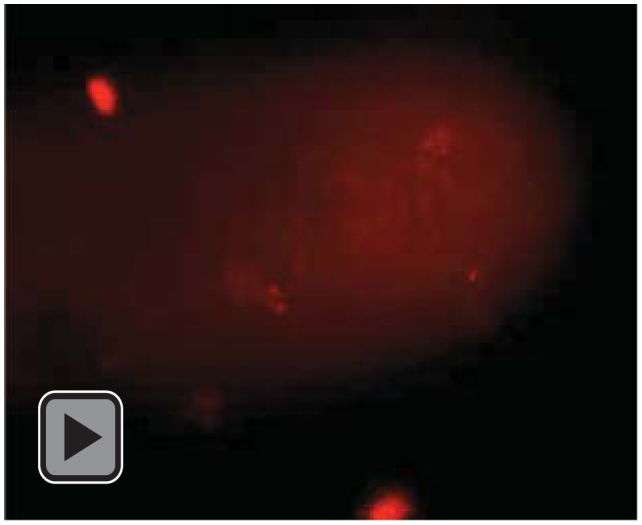
Spinning disc confocal sequential Z-axis changes in focus through a *Drosophila melanogaster* ovarian follicle. This follicle was treated with staurosporine, which has caused termination of yolk-precursor endocytosis. Initial focus is within the epithelium where dye remains in the spaces between epithelial cells. As focus moves deeper, dye-filled space between epithelium and oocyte can be seen. Note that interior to this space there are no nascent yolk spheres being produced. Click image to view video. Download video

### Patency of epithelial-oocyte gap junctions: can CaM come from epithelium?

Given the evidence that the CaM needed for signalling ongoing uptake of YPs is from the epithelial cells, it must then have some way of making the transit. While insect gap junctions had regularly been known to transmit molecules only 3kDa or smaller, in more recent reports it has been shown that, because of its elongate shape, CaM (17kDa) can also pass through insect gap junctions (Zang and Kunkel 1994; Cieniewicz and Woodruff 2008; Brwon et al. 2010).

**Figure 2.**
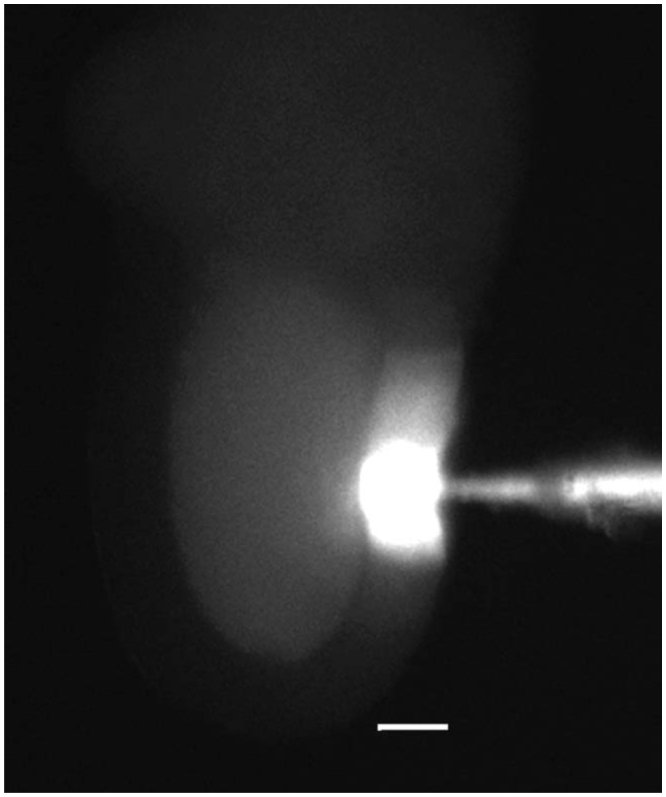
Lucifer Yellow CH iontophoretically microinjected into one epithelial cell of a *Drosophila melanogaster* ovarian follicle. Dye passed via intercellular bridges and gap junctions to surrounding epithelial cells and through open gap junctions into the oocyte. Scale bar = 30 μm. High quality figures are available online.

To demonstrate that *D. melanogaster* gap junctions between epithelial cells and oocytes were patent (open), Lucifer yellow-CH was iontophoretically introduced into individual epithelial cells (Figure 2). Lucifer yellow travelled into adjacent epithelial cells and into the oocyte.

**Table 2.**
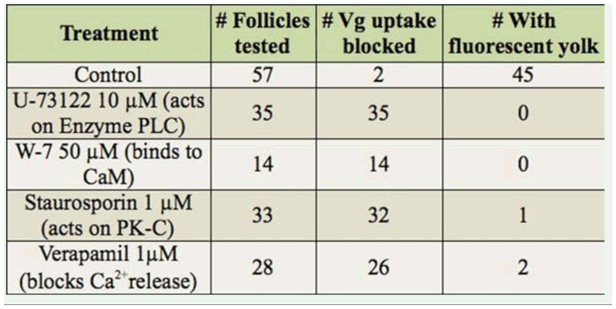
Effect of various antagonists upon active endocytic uptake of Vgs as shown by non-specific fluid phase uptake of fluorescent Dex-red dye.

**Figure 3.**
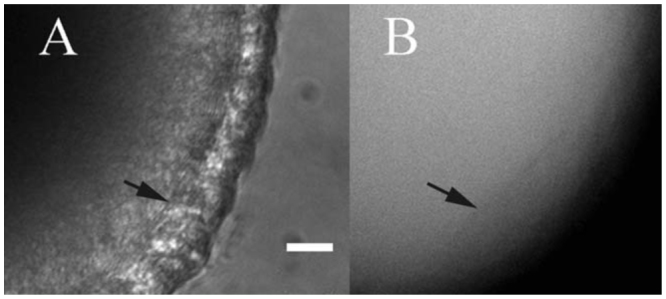
Microinjection of fluorescently labelled CaM into an oocyte of a *Drosophila melanogaster* results in the epithelial cells becoming fluorescent, confirming that the gap junctions between epithelial cells and oocyte can transport CaM. 4A, DIC, 4B fluorescence optics only. Arrows mark the inner edge of the epithelium. Scale bar = 30 μm. High quality figures are available online.

To determine if the larger CaM needed for the PLC system could pass through the gap junctions, Alexa-Fluor labelled CaM was pressure microinjected into oocytes. Figure 3A is a DIC image of a follicle microinjected with fluorescent CaM. Figure 3B is a fluorescence image of the same follicle. In both micrographs, the arrow marks the inner edge of the epithelium.

### Membrane potentials of oocytes and of epithelial cells:

The average oocyte potential in the *D. melanogaster* PSS used was -31.9 ± 0.9 mV SE, while that of epithelial cells that surround each oocyte averaged -36.5 ± 1 mV SE. Since epithelial cells are not a floating population, but rather remain attached to each specific oocyte, analysis of the data for statistical significance was made by paired *t*-test. For 54 paired measurement of oocytes and an epithelial cell attached to each specific oocyte *p* = < 0.001

### Formation of 1 to 2 μm diameter vesicles

**Figure 4.**
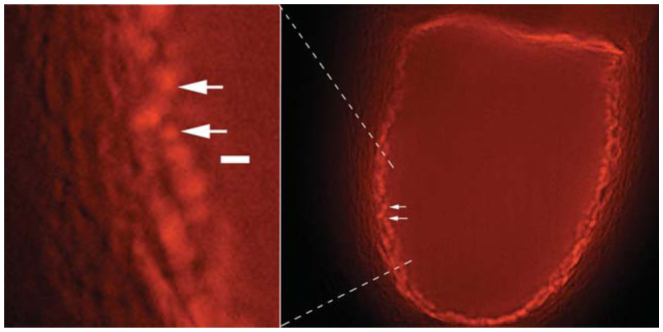
Confocal optical section of a *Drosophila melanogaster* follicle incubated in the presence of verapamil. Visible are 1–2 μm dye-filled “vesicles” that were never further internalized. In the expanded region at left a 2 μm white scale bar is shown. For convenience, it has been placed close to 1 and 2 μm “vesicles.” High quality figures are available online.

In the most favorable tissue, the interfaces between follicle cells, the most favourable tissue, peri-oocytic space and oocyte surface can be clearly seen. In antagonist-treated examples, such as is seen in Figure 4, the oocyte surface can be seen to contain numerous 1 to 2 μm diameter vesicles. However, even with long incubations of more than 2 hr, these never became internalized when any of the antagonists were present. Furthermore, they were not seen in control follicles, where such vesicles were rapidly internalized and formed into clusters. In the clusters, membrane fusion adult yolk spheres of 6.1 ± 2 were formed (Figure 1A).

## Discussion

### Is PLC the target of CaM?

In many cells active in endocytosis, an extracellular signal binds to a metabotropic membrane bound receptor. The receptor commonly activates G-protein, which in turn activates membrane bound PLC. PLC functions to convert PIP_2_ into IP_3_ and DAG (cf. (Nishizuka 1995; McCullar et al. 2007). However, as previously shown for *O. fasciatus*, in *D. melanogaster* the signal must not be extracellular, as younger follicles sharing the same ovariolar environment are not stimulated. Instead, data from above and elsewhere suggest that it is intracellular CaM of epithelial cell origin (Andruss et al. 2004), and is transferred to the oocytes via gap junctions (Waksmonski et al. 2002; Brooks et al. 2004), which permits the signal cascade to function. While CaM undoubtedly has many functions in the oocyte, what was observed in the present study was most likely an interaction between CaM and PLC. This is evidenced by the ability of W-7 and U-73122 each being able to shut down Vg endocytosis. Each of these acts in a different way to stop PLC from converting PIP_2_ into IP_3_ and DAG. W-7 binds to CaM, which can then no longer bind to PLC, while U-73122 inactivates the CaM binding site of the enzyme (Koganezawa et al. 2002; Vicencio et al. 2006). As with many antagonists/inhibitors, none of those used in the present study have been shown to have only a single target. However, when different agents produce the same results, the likelihood that the assumed target is correct increases. Thus, the data from the W-7 incubations strongly implicated CaM as the initiating signal. While G-protein is usually involved in activation of PLC, direct activation by CaM is known to occur (Pan et al. 2005). U-73122 inactivated the enzyme PLC, providing evidence suggesting the involvement of that membrane bound enzyme, as did inactivation of DAG, one of the direct products of PLC.

### Is PK-C required for endocytosis?

PK-C is known to function in endocytosis (Dabdoub et al. 2002; Gusarova et al. 2009). Treatment with staurosporine has been shown to inhibit PK-C activity and block endocytosis (Abreu et al. 2008), and it proved capable of stopping endocytic uptake in the present experiments. Both [Ca^2+^]_i_ increase and DAG are known to stimulate activity of PK-C (Dabdoub et al. 2002). Involvement of PK-C may also be inferred from the action of verapamil or CdCl_2_ (CdCl_2_ data not shown), each of which have frequently been used to prevent increase in cytosolic Ca^2+^, and each of which were found to terminate Vg uptake. Thus, Ca^2+^ release is normally required for endocytic uptake. The effect of the specific PK-C antagonist staurosporine, even when no Ca^2+^ channel-blockers were present, indicated that the target of this signal was PK-C. DAG also appeared to play a role in stimulation of PK-C, as treatment with HAG, an antagonist of DAG, terminated endocytosis. Either DAG alone or Ca^2+^ alone are known to activate PK-C. Since either inactivation of DAG or blockage Of Ca^2+^ completely stopped endocytosis of YP, it is assumed that normal levels of these were such that activation required the synergistic action of both on PK-C.

In summary, the requirement of CaM for stimulation of PLC was shown by the specific effect of W-7, an antagonist of CaM that binds to that molecule. While direct stimulation of PLC by CaM has been demonstrated in *Lillium* pollen (Pan et al. 2005), our results show only that CaM is required. Although direct interaction between CaM and PLC cannot be shown by the techniques used in the present study, the importance of PLC was shown by the effects of U-73122, which acts directly upon the enzyme by blocking the enzyme's binding sites, and by the effect of inactivation of its known products, IP_3_ and DAG. IP_3_ is known to stimulate Ca^2+^ release and verapamil and CdCl_2_, both of which prevent increase in cytoplasmic Ca^2+^ by blocking Ca^2+^ release channels, terminated formation of nascent yolk spheres. The requirement of stimulated PK-C was demonstrated by the termination of Vg uptake when the PK-C antagonist staurosporine was employed. Stimulation of PK-C by DAG was indicated by the results seen following the inactivation of DAG by HAG.

We propose that stimulation of NYS formation in *D. melanogaster* requires a signaling cascade beginning when folliclecell-produced CaM enters the oocyte via gap junctions, directly or indirectly activating membrane-bound PLC to transform PIP_2_ into IP_3_ and DAG. IP_3_ stimulates release of Ca^2+^ from internal stores, and the Ca^2+^ works in consort with DAG to stimulate PK-C. This enzyme in turn is required for endocytic uptake of YPs into NYS, clustering of NYS, and their fusion into mature yolk spheres.

### Are gap junctions and CaM needed for YP endocytosis?

There are both gap junctions and intercellular bridges (ring canals allowing passage of molecules among follicle cells (Ramamurty and Engles 1977; Woodruff and Tilney 1998; Airoldi et al. 2011). However, the present study is concerned exclusively with gap junctions that connect follicle cells to an oocyte.

That epithelial cell-to-oocyte gap junctions might play some undetermined role in regulating events during insect vitellogenesis was implied by the following observations. During *in vitro* incubation, intact follicles of *Hyalophora cecropia* were shown to avidly incorporate Vgs into yolk (Hausman et al. 1971), while nude oocytes, even when the epithelia were present in the same culture, formed no new yolk spheres (Anderson 1971). Gap junctions between the follicle epithelium and the oocytes were observed in *Locusta migratoria* (Wollberg et al. 1976), *H. cecropia* (Woodruff 1979), *Rhodnius prolixus* (Huebner 1981), and *O. fasciatus* (Woodruff et al. 1984). Of particular interest is the *D. melanogaster* female sterile mutation fs(2)A17. In females homozygous for this gene, epithelial cell-oocyte gap junctions became abnormal at the time when vitellogenesis should have begun, and no YP uptake occurred (Giorgi and Postlethwait 1985). Without yolk, eventual zygotes could not survive. Woodruff and Anderson (1984) noted that *in vitro* studies of *O. fasciatus* follicles showed the onset of epithelial cell-oocyte dye coupling occurred just prior to the formation of the first yolk spheres. They speculated that intercellular communication through gap junctions might have a role in regulating vitellogenic activity. A similar temporal link between gap junctional communication and endocytic uptake of Vgs was found in *H. cecropia* (Woodruff and Telfer 1990).

For follicles from six different orders of insects, a diffusible molecular signal able to pass through fully open insect gap junctions, but excluded from down-regulated gap junctions, was needed for the onset and continuation of endocytic uptake of yolk precursor proteins. Included were insect species with panoistic ovarioles (*Acheta domesticus*) and meroistic ovarioles of both polytrophic (*D. melanogaster, Xylocopa verginica, Actias luna*) and telotrophic (*Tenebrio molitor, O. fasciatus*) types (Waksmonski et al. 2002). Follicles with down-regulated gap junction could be rescued by microinjection of the soluble fraction of epithelial cell cytoplasm (Anderson et al. 2001; Waksmonski et al. 2002). The diffusible signal was shown to be CaM (Brooks et al. 2004).

For some of these insects, the principle site of CaM production had been shown to be in the follicle cells (Zang et al. 1994; Brooks et al. 2004), and it was speculated that CaM was transported to the oocyte via gap junctions. Furthermore, it was suggested that CaM might play a role in regulating endocytosis of Vgs (Zhang et al. 1994). These speculations were then proven correct when it was demonstrated that CaM, despite its large size of 17 kDa, and presumably because of its elongate shape (Babu et al. 1988), could pass through insect gap junctions (Brooks et al. 2004; Woodruff 2005; Cieniewicz et al. 2008) as well as those of some vertebrates (Curran et al. 2007; Cieniewicz et al. 2009). Additionally, in *D. melanogaster* it has been shown genetically by use of null-mutant germ-line cells that there is little or no CaM produced in the oocyte, and that the source of most or all of oocyte CaM is from the ovarian follicle epithelial cells (Andruss et al. 2004). However, these studies utilizing germ-line cells with non-functional CaM genes required sectioned material for immunofluorescent detection of CaM, and thus could make no determination of how follicle cell-produced CaM could reach the oocyte.

### Could CaM required for *D. melanogaster* YP-uptake come from follicle epithelial cells via gap junctions?

That gap junctions between epithelial cells were patent can be seen in the present study, in which Lucifer Yellow was iontophoretically microinjected into individual epithelial cells. *D. melanogaster* epithelial cells are linked to each other via gap junctions and in some cases via intercellular bridges (ring canals) (Woodruff et al. 1998). Thus, the injected cell and its immediate neighbours fluoresced most brightly, while the dye spread in decreasing concentration to other more distant epithelial cells. Dye also spread to the oocyte, where the fluorescence appeared much less bright due to the vastly greater volume of the ooplasm. No dye was seen in the medium, which confirmed that dye spread to the oocyte must have been through gap junctions. An elegant series of experiments demonstrated that lucifer yellow pressureinjected into *D. melanogaster* oocytes could be forced through patent gap junctions into epithelial cells. Strikingly, within 20 minutes the dye from epithelial cells had returned to the oocyte (Bohrmann et al. 1993). This return may have been driven in part by an electrical gradient, in which epithelial cells are reported here to be more electronegative than oocytes. This has proven to be the case in other insects of other orders as well (Wang et al. 1998; Adler et al. 2000; Munley et al. 2009). The experiments reported here demonstrated similar behavior for the negatively charged but larger molecules of CaM. Being forced against the electrical gradient in the gap junctions, the less mobile CaM never reached a concentration in the epithelial cells to match that of lucifer yellow. However, CaM did become visible in these cells and, like lucifer, quickly returned to the oocyte.

Epithelia around some insect ovarian oocytes have been shown to be the principle sites of CaM production far exceeding any possible oocyte production of this molecule (Zang et al. 1994; Brooks et al. 2004). Origins of CaM in other genera of insects cannot be taken as proof of a similar origin in *D. melanogaster;* however, epithelial cell origin has been genetically shown to be true (Andruss et al. 2004). The demonstration of CaM movement through the relevant *D. melanogaster* gap junctions shown here confirms that epithelial cell-to-oocyte passage of *D. melanogaster* CaM is via gap junctions. While CaM had to be forced from oocytes to epithelial cells by pressure injection, the fairly rapid return of CaM from epithelial cells to oocyte confirmed the natural movement of CaM in that direction. While CaM has been shown by immunostaining to be produced in even the youngest somatic tissue (Andruss et al. 2004), it does not begin to show in germ-line tissue until follicle cell-to-gap junctions become patent.

### Additional observations

The present experiments led to additional observations, which suggested directions for future investigation. In favorable confocal optical sections, the formation at the oocyte surface of 1–2 μm diameter vesicles was noted to have a high degree of fluorescence. These gave the appearance of the first signs of ligand binding and vesicle closure leading to actual NYS. However, no further internalization occurred even after several hours, and such vesicles were not characteristic of control follicles. In control follicles, endocytosis was rapid enough for vesicles to clearly detach from the oocyte membrane and quickly organize into deeper clusters, where they merged into mature yolk spheres. Thus, there was the suggestion that the first steps of endocytosis may have occurred without the PLC/PK-C pathway activation needed for internalization and processing.

## Dedication

This paper is dedicated to the memory of Dr. William H. Telfer, who passed away in November 7, 2010. Bill was one of the kindest and most effective mentors one could experience. His careful and limpid writing taught many of us how a scientific article should be constructed. His contributions to the field of vitellogenic oogenesis were often groundbreaking and always of value. He is greatly missed by all who had the good fortune to have known him.

## Abbreviations

CaM: calmodulin
DAG: diacylglycerol
NYS: nascent yolk spheres
PK-C: ProteinKinase-C
PLC: phospholipase-C
PSS: physiological salt solution
Vg: vitellogenin
YP: yolk protein
YPC: ovarian follicles cleared of any unincorporated YPs
